# Efficacy evaluation and mechanism study of electroacupuncture intervention in acute phase of IFP: study protocol for a randomized controlled trial

**DOI:** 10.1186/s13063-021-05632-8

**Published:** 2021-09-28

**Authors:** Yinan Qin, Lihong Yang, Man Zhang, Yang Bai, Zexin Li, Nana Zhao, Zhimei Li, Tianyu Xu, Yue Xie, Yuanhao Du

**Affiliations:** 1 First Teaching Hospital of Tianjin University of Traditional Chinese Medicine, Tianjin, 300193 China; 2 National Clinical Research Center for Chinese Medicine Acupuncture and Moxibustion, Tianjin, 300073 China; 3 Shaanxi University of Chinese Medicine, Xianyang, 712046 Shaanxi China; 4 Department of Acupuncture and Massage, Qingyang Hospital of Traditional Chinese Medicine, Qingyang, 745000 Gansu China

**Keywords:** Electroacupuncture, Idiopathic facial palsy, Acute phase, Study protocol

## Abstract

**Background:**

Previous studies had already reported the efficacy of electroacupuncture treatment for idiopathic facial nerve palsy (IFP) in a recovery phase; however, the initial use of electroacupuncture in the acute phase remains controversial. Hence, in the present study, we will add electroacupuncture intervention based on oral prednisone tablets in the acute phase of IFP and compare the clinical effects with simple oral prednisone tablets. Besides, the prognosis and safety will be evaluated. The present study aims to evaluate the clinical efficacy, prognosis, and safety of electroacupuncture combined with oral prednisone tablets in the acute phase of IFP, using surface electromyography (sEMG) to objectively observe the recovery of facial expression muscle function. In addition, the morphological changes of the facial nerve were observed dynamically by magnetic resonance imaging (MRI) in the acute phase to reveal the effect mechanism of this therapy.

**Methods:**

Randomized controlled trials will be conducted in the first teaching hospital of Tianjin University of Traditional Chinese Medicine in China from September 2020 to April 2021. The treatment will be carried out in two phases, including the acute phase and the recovery phase. Eighty IFP patients will be recruited and randomized into two groups with a 1:1 ratio. Subjects in the acute phases of the control group will be given oral prednisone tablets, based on the control group, and subjects of the experimental group will be given electroacupuncture therapy simultaneously in the acute phase. Both groups will be stopped taking prednisone tablets and turn to electroacupuncture therapy in a recovery phase. Patients in the experimental group will receive treatment at least 6 times in the acute phase and both groups will receive treatment 9 times in the recovery phase, and there will be a follow-up period of 15 days after the treatment. The primary outcome is supposed to be related to the changes observed in the Sunnybrook (Toronto) Facial Grading System (SFGS) on the baseline and day 30 after the onset. Secondary outcome measures will include House-Brackmann Facial Nerve Grading System (H-B), measurement of Numerical Rating Scale (NRS), Facial Disability Index scale (FDI, including physical function subscore, FDIP, and social functioning and well-being subscore, FDIS), surface electromyogram (sEMG), and magnetic resonance imaging (MRI). Adverse events will be recorded for safety assessment.

**Discussion:**

The results of this trial will allow the present study to compare the difference in efficacy and prognosis between the strategy of combined electroacupuncture in the acute phase and only oral prednisone tablets. The findings from this trial will be published in peer-reviewed journals.

**Trial registration:**

CHICTR ChiCTR2000038472. Registered on 23 September 2020

## Background

### Epidemiology and current management

Facial palsy is a common clinical disease characterized by facial nerve palsy. It leads to dysfunction of the facial expression muscles. The most common form of facial nerve palsy is idiopathic facial palsy (IFP), also called Bell’s palsy, which accounts for more than half of all facial nerve palsy [1]. The terminology of the disease is based on the Scottish anatomist Sir Charles Bell (1774 to 1842), as he is the first person who recognized that idiopathic facial palsy is caused by involvement of the seventh cranial nerve [2]. At present, there are as high as 25/10,000 people who suffer from Bell’s palsy in the age group of 30–45. Moreover, 1 in 60 individuals gets affected over the course of their lifetime [3, 4]. The clinical manifestations of IFP are related to motor dysfunction of the facial expression muscles and manifested as shallow forehead lines, incomplete closure of the eyelids, enlarged palpebral fissures, inability to frown, spontaneous tears, oblique mouth drooping, bulging cheeks leaking air to one side, difficulty in chewing and speaking, etc. [5]. In severe cases, if the sensory nerve fibers are damaged, there will be auditory dysfunction, facial muscular weakness, disturbance of taste, and disorder of salivary secretion [6].

It has been proved that steroids can certainly reduce facial nerve edema. For IFP patients (over 16 years old), a supplement of oral steroid prescription within 72 h of the onset of symptoms is strongly recommended as A-level evidence in the 2013 American Otorhinolaryngology Head and Neck Surgery Clinical Practice Guidelines [7].

### Rationale for the use of intervention

Generally, IFP is a kind of self-limiting disease, and the recovery of Bell’s palsy is usually optimistic. However, there are still 10 to 30% of patients who are observed with residual weakness, asymmetry, or else impaired function [5, 8]. Although this disease is not life-threatening, the facial nerve innervates a total of 23 paired muscles as well as the orbicularis oris. Usually, facial expressions play an indispensable role in interpersonal communication. Therefore, the disease may have a more or less significant impact on psychological [9, 10].

The pathogenesis of IFP is still uncertain. There are two possible mechanisms which probably develop this disease. First, the facial nerve itself is damaged. On the one hand, the spasm of the blood vessels related to the local nutrient nerves caused by wind and cold leads to nerve ischemia, edema, and compression. On the other hand, the facial nerve is infected by rheumatism factor or virus. Second, other peripheral factors can also lead to facial nerve injury. The periostitis in the foramina stylomastoideum causes facial nerve compression or blood circulation disorder or facial nerve edema surrounding the medial temporal area. However, insufficient space for nerve expansion in the bone canal may result in compartment syndrome caused by edema, ischemia, and loss of nerve function [11, 12]. In the incipient of pathological changes related to IFP, the main manifestations are edema and demyelination of the facial nerve, while in the terminal stage, they are related to axonal degeneration and nerve atrophy. The most significant parts are the foramina stylomastoideum and facial canal [12].

However, based on the above pathogenesis, facial nerve edema makes the compression state formed in the facial nerve canal and the blood vessels simultaneously. The circulatory disorder caused by compression makes it difficult to achieve superior blood drug concentration when the drug acts on the local target. This ultimately results in the weakening of the anti-edema and anti-inflammatory effects. Currently, facial nerve decompression (FND) has also been suggested with a feasible surgical technique theoretically. However, there is not enough evidence which can prove if FND intervention is beneficial [13–15]. Therefore, timely intervention and resolution of edema for the acute phase are essential to achieve the best therapeutic effect.

### Rationale for the trial design

Acupuncture as a traditional Chinese medicinal therapy has been widely used to treat IFP [16–20]. Moreover, it is strongly recommended as level I evidence in the evidence-based acupuncture therapy reported by Chinese scholar Dr. Du Yuanhao [21]. Further, recent studies have confirmed the clinical efficacy of acupuncture and electroacupuncture. However, in past few years, controversies have emerged about the use of electroacupuncture in the acute phase [18, 22–26]. On the one hand, some studies and their experiences have proved that electroacupuncture therapy can repair damaged nerves, promote neurological rehabilitation, shorten the disease course, and improve the curative effect [27, 28]. On the other hand, other studies and their experiences revealed that it is more likely to aggravate facial nerve edema and bioelectric conduction disorders.

Therefore, we designed a prospective randomized controlled trial for patients with IFP who are in the acute phase to compare the effects of electroacupuncture intervention combining oral prednisone tablets with simple oral prednisone tablets. The combination of these two treatments not only helps in protecting the rights and interests of patients but also avoids treatment delays due to the bias involved in the research design [29]. The present study aims to evaluate the clinical efficacy prognosis and safety of electroacupuncture combined with oral prednisone tablets in the acute phase of IFP. In addition, we will also use surface electromyography (sEMG) and MRI for testing. These tests will aim to observe the efficacy from an objective perspective and reveal the effect mechanism of this therapy. To ensure the completeness of treatment, in the recovery phase of IFP, both groups adopt currently accepted treatment methods.

The double-blind, placebo-controlled trial is the golden standard to assess the therapeutic effect. However, the two treatment methods used in the present study are easily distinguished. Thus, in this study, we did not include the blinding method. However, in the present study, we used a blind method for evaluation. Further, efficacy assessments will be performed by the third parties, and they were unaware of the grouping situation.

## Method/design

### Study design

**Fig. 1:**
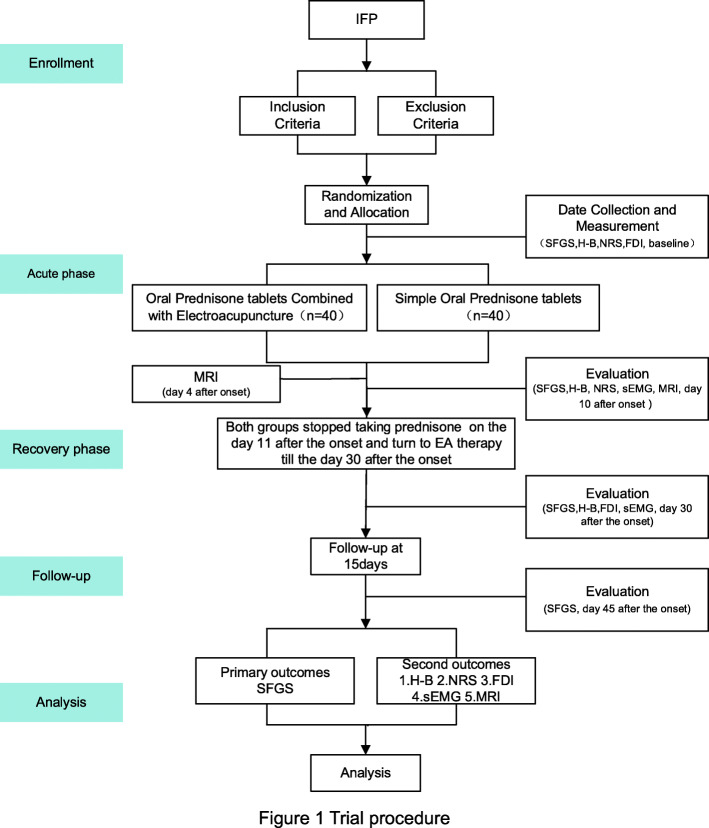
Trial procedure

The proposed study is a parallel-group superiority randomized controlled trial with a 1:1 allocation ratio and a blind method for evaluation. We will compare the effects of two available treatment options for patients with IFP in the acute phase. The study will include the treatment phase and follow-up period. Trial procedures are described in Fig. 1, whereas Table 1 illustrates the trial schedule. In total, 80 IFP patients will be recruited and randomized into one of two groups with a 1:1 ratio of the experimental group (oral prednisone tablets combined with electroacupuncture) and the control group (simple oral prednisone tablets). In the present study, patients will be recruited from the First Affiliated Hospital of Tianjin University of TCM from September 2020 to March 2021. All patients will be required to provide written consent to participate in the study. This study is approved by the Ethics Committee of the First Affiliated Hospital of Tianjin University of TCM (TYLL2020[K]049). Furthermore, to carry out this study, we registered the study in the Chinese Clinical Trial Registry (ChiCTR2000038472).

**Table 1:** Schedule of enrollment, intervention, and assessments of this study protocol

	Study period					
	Enrollment	Treatment				Follow-up
Time point	Day 0	Day 4 after the onset	Day 10 after the onset	Day 11 after the onset	Day 30 after the onset	Days 31–45 after the onset
**Enrollment**						
Eligibility screen	●					
Informed consent	●					
Date collection	●					
Measurement	●					
Randomization	●					
Allocation	●					
**Intervention**	Acute phase			Recovery phase		Follow-up period
Control group	Oral prednisone tablets			Stop taking prednisone and turn to electroacupuncture therapy		No intervention
Experimental group	Oral prednisone tablets combined with electroacupuncture					
**Assessment**						
SFGS	●		●		●	●
H-B	●		●		●	
NRS	●		●		●	
FDI	●				●	
MRI		●	●			
sEMG			●		●	
Adverse event	●	●	●		●	

### Participants

#### Participants criteria

The following are the inclusion criteria for patient selection:

1. Patients with unilateral onset within 3 days
2. Between the ages of 18 and 78, both gender
3. Being able to actively cooperate with the doctor and sign the consent form for voluntary acupuncture treatment

#### Exclusion criteria

The following are the exclusion criteria for patient selection:

1. The participants are excluded if previously they had suffered from recurrent facial nerve palsy, central facial palsy, facial palsy caused by Lyme virus and simplex virus (Hunt facial palsy), and traumatic facial nerve injury.
2. Participants with severe cardiovascular and cerebrovascular diseases, cancer, and liver and kidney dysfunction.
3. Participants during pregnancy and lactation.
4. Participants who are receiving other antiviral therapy and corticosteroids therapy for other diseases simultaneously.
5. Patients with pacemakers, silver clip ligation of aneurysm, and metal foreign bodies or metal prostheses in the body.
6. Claustrophobic.

### Recruitment, randomization, allocation concealment, and blinding

#### Recruitment

Patients will be recruited from advertisements via a notice board in the clinics of acupuncture and moxibustion department, the First Affiliated Hospital of Tianjin University of TCM and WeChat group dissemination, etc.

#### Random and allocation of hidden methods

The method of grouping is to use the random numbers generated by computer software (IBM SPSS 22.0); numbers 1–80 at first and then randomized grouping by using random number generator (fixed value is 826): 0 = experimental group and 1 = control group. The abovementioned are operated by the person who is responsible for the statistics. According to the result of the grouping, we will make the random card and sealed each card in an opaque envelope, which will be kept by a responsible person. Once a participant met the inclusion criteria and gave consent to our research assistant, the next envelope in the order will be opened, and the treatment methods will be assigned.

#### Blind method

This study will use the blind method for evaluation. Efficacy assessments will be performed by third parties. They are blinded for the randomization sequence and treatment allocation. Blinding statistical analysis will be used in the data summary stage, that is, statistical analysts will be unaware of the randomization sequence and treatment allocation.

### Interventions

The treatment will be conducted in two phases, including the acute phase and the recovery phase. Subjects in the acute phase of the control group will be given oral prednisone tablets, whereas subjects in the recovery phase will be stopped taking prednisone tablets and turn to electroacupuncture therapy, subjects of the experimental group will be given electroacupuncture therapy simultaneously based on the method used in the control group in the acute phase, and the treatment in the recovery phase will be the same as that in the control group.

The physician who will perform the acupuncture treatment had at least 5 years of practicing experience before the implementation of the study. We will conduct unified training for the acupuncturists who implement the treatment to ensure the consistency of the trial implementation. Acupuncture will be performed by specialists in traditional Chinese medicine, and the protocol will follow the details of the Standards for Reporting Interventions in Clinical Trials for Acupuncture 2010 checklist [30] as shown in Tables 2 and 3.

**Table 2:** Details of acupuncture intervention

Description		
1. Acupuncture rationale	1a.) Style of acupuncture	Traditional Chinese medicine.
	1b.) Rationale for treatment	Acupuncture for the treatment of facial palsy has been strongly recommended as level I evidence in the evidence-based acupuncture therapy compiled by Chinese scholar Dr. Du Yuanhao. It has also been proved to be safe and reliable in practice.
	1c.) Extent to which treatment was varied	There is no individualized part in the acupuncture process.
2. Details of needling	2a.) Number of needle insertions per subject per session	Acute phase: 6 times at least (experimental group). Recovery phase: 9 times (both groups).
	2b.) Names of the insertion points (uni- or bilateral)	Acute phase: SJ17, GB2, GB14, EX-HN4, ST2, SI18, ST4, LI4, and (affected side). Recovery phase: GB14, EX-HN4, ST2, SI18, ST4, EX-HN16, LI4, and *Ashi* point (affected side).
	2c.) Depth of insertion	10–20 mm (exact depth shown in Table 3).
	2d.) Response sought	*Deqi*.
	2e.) Needle stimulation	Simultaneous intervention of electric stimulation and manual stimulation (exact details are in Table 3).
	2f.) Needle retention time	30 min (from the last needle insertion to the stopping of the treatment).
	2g.) Needle type	0.25 mm (diameter) × 40 mm (length) disposal needle (Huatuo Acupuncture, Suzhou, China).
3. Treatment regimen	3a.) Number of treatment sessions	6 times at least in the acute phase and 9 times in the recovery phase, during the 30-day test period, no less than 15 treatments in total.
	3b.) Frequency and duration of treatment sessions	During the acute phase of 10 days except for Sunday, once a day, and 3 times a week during the next 20 days of the recovery phase.
4. Other components of treatment	4a.) Details of other interventions administered to the acupuncture group	No other interventions.
	4b.) Setting and context of treatment	Inform patients that they will receive electroacupuncture or oral prednisone tablets in the acute phase, but both groups will be treated with the same acupuncture regimen after the assessment in the acute phase.
5. Practitioner background	5.) Description of participating acupuncturists	The physician performing acupuncture treatment has at least 5 years of practicing experience.
6. Control or comparator interventions	6a.) Rationale for the control or comparator in the context of the research question	Non-acupuncture control is used as a control, and prednisolone tablets are recommended in the guideline.
	6b.) Precise description of the control or comparator	The prednisolone tablets control group forms a positive control and completes the evaluations during the 10th day after onset.

**Table 3:** Acupoints and needle insertion procedures

Acupoints	Direction	Depth (mm)
SJ17 (Yifeng, affected side)	Obliquely upward between the styloid and mastoid	10 mm
GB2 (Tinghui, affected side)	Perpendicular to the skin	10 mm
GB14 (Yangbai, affected side)	Horizontally toward the geisoma	20 mm
EX-HN4 (Yuyao, affected side)	Horizontally toward GB14 (Yangbai)	10 mm
EX-HN16 (Qianzheng, affected side)	Obliquely toward the mouth	10 mm
ST2 (Sibai, affected side)	Obliquely toward the pupil at an angle of 75°	10 mm
SI18 (Quanliao, affected side)	Perpendicular to the skin	15 mm
ST4 (Dicang, affected side)	Perpendicular to the skin	15 mm
LI4 (Hegu, affected side)	Perpendicular to the skin	20 mm
*Ashi* points (around the orbicularis oculi and orbicularis oris muscle, affected side)	Horizontally with low angle	10 mm

### Control group

#### Acute phase (from enrollment to day 10 of onset)

Patients in this phase will be given oral administration of prednisolone tablets (25 mg once daily).

#### Recovery phase (from day 11 to day 30 of onset)

Patients in this phase will stop taking prednisone tablets and will be provided with electroacupuncture therapy in the recovery phase. The exhaustive methods of electroacupuncture intervention are as follows.

**Fig. 2:**
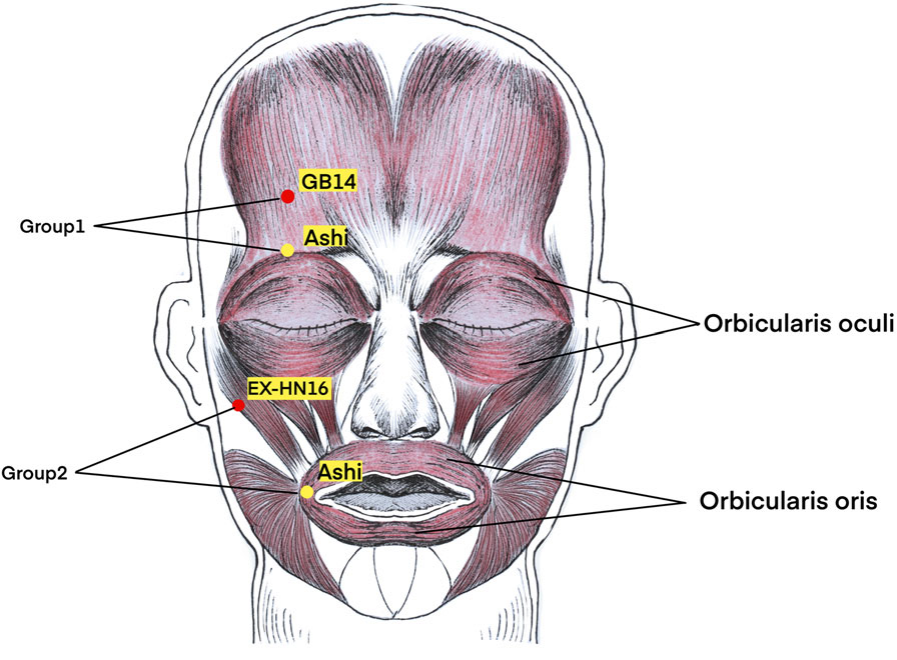
The selection of acupoints in electroacupuncture therapy and the location of *Ashi* points (negative electrode as shown in red, positive electrode as shown in yellow, two groups in total)

The patients will receive acupuncture treatments in the recovery phase for 9 times. The acupoints used in this study are Yangbai (GB14), Yuyao (EX-HN4), Sibai (ST2), Quanliao (SI18), Dicang (ST4), Qianzheng (EX-HN16), and Hegu (LI4). Furthermore, *Ashi* points will be selected around the orbicularis oculi and the orbicularis oris muscle. The acupuncture needles will be inserted horizontally with a depth of 10 mm to elicit the sensation of *Deqi* sensations. The patients will receive electroacupuncture therapy at the Yangbai (GB14) and *Ashi* point around orbicularis oculi muscle and Qianzheng (EX-HN16) and *Ashi* point around the orbicularis oris muscle (20 Hz, sparse waves), and the needles will be kept for 30 min (three times a week). The specific details about the electroacupuncture therapy and the location of *Ashi* points are shown in Fig. 2.

### Experimental group

#### Acute phase (from enrollment to day 10 of onset)

Based on the method of the control group, patients in this phase will be combined with electroacupuncture intervention. The medicinal method will be as same as that in the control group. The exhaustive methods of electroacupuncture intervention are as follows.

The experimental group will receive acupuncture treatments in the acute phase for at least 6 times. The acupoints used in this phase are Yifeng (SJ17), Tinghui (GB2), Yangbai (GB14), Yuyao (EX-HN4), Sibai (ST2), Quanliao (SI18), Dicang (ST4), and Hegu (LI4) on the affected side. Then, patients will receive electroacupuncture therapy at the Yifeng (SJ17) (negative electrode) and Tinghui (GB2) (positive electrode) (2/10 Hz, Dilatational wave). The needles will be kept for 30 min (once daily except for Sunday).

#### Recovery phase (from day 11 to day 30 of onset)

The treatment of the experimental group in the recovery phase is as same as that in the control group.

### Outcomes

In order to reduce the bias of experimental results, the following outcomes will be assessed by an independent outcome evaluator. Before the beginning of the trials, we will conduct a training session for the outcome evaluator. Further, we conducted online meetings via Tencent Meeting due to the ongoing COVID-19 pandemic.

The outcome evaluators will be performed at four time points including the baseline, on day 10 after the onset, on day 30 after the onset, and on day 45 after the onset (follow-up period).

### Main outcome indicators

The primary evaluation index in the trial will be related to the change of the scores involved in the Sunnybrook (Toronto) Facial Grading System (SFGS) on the baseline and day 30 after the onset.

#### Sunnybrook (Toronto) facial grading system

The Sunnybrook Facial Grading System is a composite of three domains (face at rest, voluntary motion, synkinesis), three facial regions at rest (eye, cheek, mouth), and five facial regions in voluntary motion with or without synkinesis (brow lift, gentle eye closure, snarl, open mouth smile, and lip pucker). Within each domain and facial region, there are three to five levels. The system will generate a composite score that describes the overall static and dynamic condition of the face [31].

### Secondary outcome

The secondary outcome was related to the different scales include the following.

#### House-Brackmann facial nerve grading system

The House-Brackmann Facial Nerve Grading System was introduced by John W. House and Derald E. Brackmann. It can evaluate the patient’s facial nerve function status and classify the patient’s facial nerve damage integrally. The grading system is easy to understand and applied quickly to clinical practice relatively [32]. The measurements will be performed within the baseline, day 10 after onset, and day 30 after onset. The number of cases reaching grade 1 on the 30th day after the onset of illness also will be counted.

#### NRS

The NRS uses numbers 0–10 to represent the degree of pain. The basic method is to divide a straight line into 10 equal parts, and the two ends of the line are numbered “0” and “10,” whereas 0 means no pain, 10 means severe pain which is unbearable, 1–3 means mild pain, 4–6 means moderate pain, and 7–9 means severe pain. The measurements will be performed within the baseline and day 10 after onset.

#### FDI

FDI is divided into two parts. FDIP is an evaluation of physical function, including eating, drinking, gargle, pronunciation, and tearing eyes; FDIS is mainly for the evaluation of social and psychological functions, including the quality of sleep after illness and the attitude of communicating with other people. The measurements will be performed within the baseline and day 30 after onset.

#### sEMG

Facial EMG has been regarded as a standard diagnostic tool for evaluating the mimetic muscles, and sEMG has proved to be a valuable, non-invasive tool in many researches and clinical applications [33]. We use this method to try to objectively reflect the changes involved in muscle electrical activity before and after treatment. The measurements will be performed within day 10 after onset and day 30 after onset.

#### MRI

MRI examination is to observe the morphology of the facial nerve of the temporal bone and the transverse diameters of the facial nerve mastoid; second, the genu and tympanic segments will be considered as the outcome indicators. The measurements will be performed within day 4 after onset and day 10 after onset. The MRI examination (SIEMENS MAGNETOM Skyra 3.0T MRI Scanner using head matrix coils) protocol for this study sequence is as follows: T1 three-dimensional magnetization-prepared rapid gradient-echo imaging with the following parameters: 256 × 256 mm field of view (FOV), repetition time/echo time (TR/TE) of 2000.00/1.97 ms, 192 sections of 1.0 mm thickness, with a voxel size = 1.0 × 1.0 × 1.0 mm, flip angle 20°, and acquisition time of 4 min, 40 s.

### Sample size estimation

According to the previous studies [34], the difference between the SFGS score before and after treatment in the oral administration of prednisolone tablets group was 32.3, with a standard deviation of 8.1. The SFGS score before and after treatment for oral administration of prednisolone tablets combined with electroacupuncture intervention group reached up to 38.3, and the standard deviation was recorded 1. Obtained results were compared with the means of two independent samples. This study was a prospective study with two groups and the sample size was 1:1. The sample size was calculated using the formula $n=\frac{\left({Z}_{\alpha }+{Z}_{\beta}\right)2\times \left(1+1/k\right){\sigma}^2}{\delta^2}$, with a test efficiency of 80%, a beta of 0.2, and an alpha of 0.025. The results show that the number of samples in each group was not less than 36. Considering the problem of missing samples or failing to follow-up, the sample size will be increased by 10%. This means that the minimum sample size of each group will be 40 patients. Finally, the total sample size of the two groups should not be less than 80 patients.

### Adverse events and solutions

Once an adverse event appears, the study team should report to the Ethics Committee and the responsible units within 24 h and record the serious adverse events in the original data in detail. Original data include the following: the time of occurrence of the adverse event, symptoms, signs, its occurrence related to the acupuncture treatment or not, and the details of the corresponding treatment. follow-up time, duration of the adverse event, and the combined medication should be recorded in detail so as to analyze the correlation between adverse events and the trial protocol, and then patients should be taken to the relevant departments immediately to accept the necessary treatment. According to the severity of the adverse event or the patient’s personal decision will be left on the researchers whether to suspend the trial or only the patient to withdraw from the trial.

### Statistical analysis

All statistical analysis will be carried out by the professionals using the SPSS (version 22.0) software. The statistical analysts will be unclear about the specific grouping content of the trial. In the present study, we will use a full analysis set (FAS) and per-protocol set (PPS), and the final conclusion will be based on the results of FAS. The full analysis set (FAS) is based on therapeutic intent. Patients who participate in the randomization but only have received 1-time treatment will also be included in the analysis. In the FAS analysis, for patients who do not complete the trial, the main indicators missed will be derived from the last observation carried forward (LOCF). All statistical analysis will be performed by bilateral differential test, with a *P* value < 0.05 considered statistically significant. Continuous variables will be presented as mean ± standard deviation (normally distributed data) or medians and ranges (non-normally distributed data). For the balance of basic values, analysis of variance or chi-square test or rank sum test will be used. For the primary and secondary outcomes, the analysis of variance will be used. If confounding factors may have an impact on the results, then the co-variance analysis will be used. Further, we will repeat measurements of the same outcome index of the same group at different time points. Changes involved in the rates before and after treatment in each group will be measured by the chi-square test or non-parametric test. The paired *t* test will be used to compare the differences between the groups before and after treatment.

### Quality control

1. This study will use a randomized control design. The randomization protocol will be carried out by the management team of the study who do not participate in the project to ensure the biases involved in the random allocation and control.
2. This study will strictly limit the inclusion criteria and formulate clear inclusion and exclusion criteria to control biases.
3. This study will use a blind method to ensure the reliability of the research results. Outcome evaluators and statistical analysts will all be blind to the randomization sequence and treatment allocation.
4. During the study, we will use sEMG to avoid bias due to its objectivity and use MRI to view the morphological changes of the facial nerve from a more objective perspective.

### Quality assurance

Before the launch of the study, we will conduct a unified training for the participants involved in the implementation. Key training will be carried out on the project implementation plan and standard operation procedures (SOPs), so that each clinical researcher will be familiar with the research process and specific implementation rules which ensures the reliability of clinical research conclusions. Furthermore, we will also explain the operation method of acupuncture at the Yifeng (SJ17) acupoint and the Tinghui (GB2) acupoint.

### Data collection and management

Paper CRFs will be used to record all the first-hand clinical trial data of subjects. Researchers should ensure recording of the data is timely, complete, accurate, and true.

During the trial, a monthly data inspection will be needed. Researchers will follow the rule of confidentiality for the trial. The data obtained from the completed CRF is recorded into the database established by Microsoft Excel for data management. After the implementation of the statistical process, the content of the CRF will no longer be modified further. After obtaining the primary data which will be kept for 3 years, non-research team members will not be allowed to browse and borrow the data, outcomes, and analyses to the relevant parties, including the fund regulator, trial participants, trial registries, and journals.

### Plans to promote participant retention and complete follow-up

Participants will be reminded by the study investigators on every consultation time and will received notifications using the phone call and online instant messaging application “WeChat.”

The study team will educate the subjects about the development trend of disease when they enrolled for the study. Furthermore, the study team will provide acupuncture treatment and oral medicine for free. And to ensure that the treatments will be administered consistently, the acupuncturists which will be assigned have at least 5 years of clinical experience in acupuncture practice.

### Data monitoring and auditing

There is no additional data monitoring committee. Instead, the study investigators will monitor the survey data in the database on a regular basis for any issues. If there are any issues, they will be discussed at the weekly study team meetings. Once any issues are detected by study investigators, those issues will be reported strictly to the principal investigators immediately, who will then escalate them to the Ethics Committee of the First Teaching Hospital of Tianjin University of TCM when appropriate.

### Informed consent

Before conducting a conversation with the subject, the researcher will explain the entire content of the informed consent form to the subject in a simple and understandable language. The content will include the purpose of the test, the test process, the possible benefits and risks, and the rights and obligations of the subjects. After the subjects learned the details, they decided to voluntarily participate in this clinical trial and voluntarily sign the “informed consent.” They will be having the right to withdraw at any given time and at any stage of the trial without discrimination or retaliation. This trial will comply with the rights and obligations of the subjects as stipulated in the Declaration of Helsinki.

### Post-trial care

This study will not provide any post-trial care.

### Protocol amendments

Participants may request to discontinue participation in the study at any time. In addition, if there are complications that affect the patient’s condition during the treatment, we will modify the intervention.

If the protocol changes during the implementation of the study, researchers will communicate the important protocol modifications (e.g., changes to eligibility criteria), and paper CRFs will be used to record all the first-hand clinical trial data of the subjects. Researchers should ensure recording of the data is timely, complete, accurate, and true.

### Confidentiality

This study follows the ethical principles set out in the “Helsinki Declaration” and the various laws and regulations related to clinical research in China. During the study, patients will receive treatments such as electroacupuncture. In addition, patients will receive free sEMG examination, MRI examination, and various evaluation tests. There is a minor chance of fainting, bleeding, hematoma, or infection during the study. If such situations appeared, we will treat the patient appropriately and record it in time**.**

## Discussion

Bell’s palsy, is the most frequently encountered type of peripheral facial palsy. It is an acute and self-limiting form of inferior motor neuron palsy. However, some part of patients cannot recover to the condition before the illness. The only prognostic factor of IFP is the state of the facial nerve 1 week after the onset [35], and the treatment in the acute phase of the disease largely determines the fate of FN and the recovery of facial function. Therefore, finding an effective treatment 1 week after the onset of illness is essential for an optimistic recovery. Previous studies have proved that steroids can relieve facial nerve edema. However, due to the compression state formed in the facial nerve canal in the acute phase, local blood circulation can be impaired. This may prevent oral steroids from reaching effective blood concentration in the facial nerve canal lesion, thereby affecting the efficacy and becoming a bottleneck in treatment. Stimulating the facial nerve canal through electroacupuncture locally may promote blood circulation in the facial nerve canal and relieve inflammatory edema. At the same time, improvement in the local microcirculation creates favorable conditions for the drug to reach effective blood concentration.

Worldwide, IFP is considered to be one of the most suitable indications for acupuncture treatment. Acupuncture has become the main treatment for IFP and exhibited significant effect according to the results of preliminary systematic evaluation and clinical studies. Electroacupuncture therapy is a kind of stimulation which directly acts on the target nerve through a combination of local acupuncture stimulation and electrical stimulation. Whether electroacupuncture can be used in the acute phase is still a subject of controversy. However, in the present study, the implementation of electracupuncture was scientific, detailed and innovative. Combining the theories of traditional acupuncture with modern anatomy, we select the Yifeng (SJ17) and Tinghui (GB2) electroacupuncture points to intervene in the acute phase of IFP; it will act directly around the target nerve.

According to the modern anatomical body surface projection positioning, Yifeng (SJ17) is close to the stylomastoid foramen (SMF), where the junction of the intracranial and intratemporal of the facial nerve is present. Tinghui (GB2) is close to the arborization of the extratemporal facial nerve which typically begins within the substance of the parotid gland [11]. The above two acupuncture points are markedly close to the facial nerve. Acupuncturing these two acupoints to a certain depth can directly stimulate the area around the FN. Stimulation frequency is one of the important parameters of electroacupuncture. Choosing different frequencies has completely different positions and effects. In the acute phase of the present study, the dilatational wave with a frequency of 2/10 Hz of electroacupuncture is used, which can relax and contract muscles rhythmically, accelerate blood circulation and lymphatic circulation, regulate the nutrient metabolism of tissues, and eliminate inflammation and edema [36]. Stimulating at the neuromuscular junction induced by acupuncture can lead to afferent sensory networks and activate the paraventricular nucleus of the hypothalamus to secrete vasopressin, corticotrophin-releasing hormone, and then, adrenocorticotrophic hormone (ACTH) is released into the bloodstream. ACTH then activates the zona fasciculate of the cortex of the adrenal glands to release glucocorticoids [37–39]. Previous studies have shown that [40], at the later stage of treatment, the number of Nissl bodies which represent the survival of neurons has returned to pre-injury level. Further, on the 14th and 21st days, more myelinated axons and fewer inflammatory cells were found in the electropuncture group. However, in the recovery phase, the sparse wave with a frequency of 20 Hz of electroacupuncture is used. Continuous stimulation at this phase will cause repeated local muscle contractions, which is helpful for local blood circulation of the face. Studies have shown that electroacupuncture invention can also help repair damaged nerves and promote their recovery of the nerve recovery phase [41].

In the design of clinical trials, the selection of appropriate outcome indicators is critical to evaluate the clinical efficacy. Nowadays, most evaluation methods related to facial nerve function of patients mostly rely on neurological function scales and electrophysiological data and cannot easily observe changes in symptoms and condition. Hence, in the present study, we not only used a series of evaluation scales certified by international guidelines, but also used more intuitive detection technologies such as surface electromyography and MRI, which can evaluate the clinical efficacy before and after the treatment. Surface electromyography can more objectively reflect the recovery of the patient’s facial muscle function, whereas MRI can more directly reflect the recovery of facial nerve edema from the morphology. Moreover, we used NRS to evaluate the degree of pain behind or around the ear, although this symptom did not observe in all the patients. However, according to the epidemiological survey on IFP in the early years, the incidence of pain behind or around the ear is 63% [42], which often affects the recovery of the condition. Therefore, we conducted a statistical analysis and analyzed the degree and duration of pain.

However, this study also has few limitations. First, due to the particularity of acupuncture, we could not blind acupuncturists and patients; however, only evaluators and statisticians were blinded as much as possible. Second, the time interval between follow-up visits was too long, with no other follow-up points set due to the limited research funding and time. Part of the reason was related to the patient’s complex compliance problem. Most patients ended the cycle of follow-up observation in advance after meeting the remarkable improvement of acupuncture treatment. Nevertheless, this research will provide reliable evidence of efficacy and safety for the clinical treatment related to IFP.

### Trial status

Protocol version number and date: V1.1, dated 14 February 2021. The first participant was enrolled on 28 September 2020. Recruitment will be completed on 30 April 2021.

### Supplementary information

The study protocol has been reported in accordance with the Standard Protocol: Recommendations for Interventional Trials (SPIRIT) guidelines.

## Data Availability

The datasets generated and/or analyzed are available from the corresponding author upon reasonable request.

## Abbreviations

IFP: Idiopathic facial palsy
SFGS: Sunnybrook (Toronto) Facial Grading System
H-B: House-Brackmann Facial Nerve Grading System
NRS: Numerical Rating Scale
FDI: Facial Disability Index scale
sEMG: Surface electromyogram
MRI: Magnetic resonance imaging
FP: Facial palsy
FND: Facial nerve decompression
SAE: Serious adverse event
FAS: Full analysis set
PPS: Per-protocol set
LOCF: Last observation carried forward
SOP: Standard operation procedure
ACTH: Adrenocorticotrophic hormone
